# The Success of Treatment Free Remission in Chronic Myeloid Leukaemia in Clinical Practice: A Single-Centre Retrospective Experience from South Africa

**DOI:** 10.1155/2023/2004135

**Published:** 2023-07-28

**Authors:** Siddeeq Hoosen, Irene Mackraj, Nadine Rapiti

**Affiliations:** ^1^University of KwaZulu-Natal, School of Laboratory Medicine and Health Sciences, Durban, South Africa; ^2^National Health Laboratory Service, IALCH, Department of Haematology, Durban, South Africa; ^3^King Edward VIII Hospital, Department of Clinical Haematology, Durban, South Africa

## Abstract

**Introduction:**

Chronic myeloid leukaemia (CML) management has evolved from a disease once considered to be incurable just over 2 decades ago to that of one of a “functional cure” as defined by the sustained molecular response on stopping tyrosine kinase inhibitor(TKI) therapy. The next goal of CML management has been treatment-free remission (TFR). The past 4 years have seen much international data on TFR attempts in CML in clinical practice. However, Africa as a continent has lagged behind the rest of the world, in keeping up with the latest trends in CML management, and so this study aims to address this gap by assessing the outcome of TFR in CML in a single centre in South Africa (SA).

**Methods:**

We conducted a retrospective cohort study in 12 CML patients in the chronic phase to assess the success of TKI discontinuation. The patients were treated in King Edward VIII Hospital (KEH), a tertiary, academic hospital in KwaZulu-Natal, South Africa, and the study period was from June 2020 to May 2022. Patients included had to have been on TKI therapy for a minimum of 5 years and achieved a deep molecular response (DMR) for a minimum period of 3 years.

**Results:**

The overall TFR cohort showed a success rate of 75% at a median follow-up of 12 months. All patients who failed TFR, defined as a loss of major molecular remission (MMR), failed within 6 months of stopping TKI therapy. All patients who failed TFR regained DMR after retreatment with TKI, with no disease progression reported. The only factor influencing the success of TFR was the total period of TKI therapy.

**Conclusion:**

Despite our study having a small cohort of patients, this study demonstrated that TFR in CML is an attainable goal, even in a resource-limited setting.

## 1. Introduction

The advances in the diagnosis of chronic myeloid leukaemia (CML) have led to the development of precision medicine for CML patients. The discovery of specific genetic mutations using various molecular laboratory techniques has improved diagnostic accuracy and timing [1]. This allows for directed therapy and closer disease monitoring, paving the way for earlier interventions and also the development of standardised guidelines for disease management.

CML is one of the common myeloproliferative disorders. CML was previously noted to have a triphasic disease course with an average life expectancy of around 5 to 7 years [2]. The discovery of the *BCR-ABL1* gene rearrangement [3–5] led to the development of targeted therapy with tyrosine kinase inhibitor (TKI) therapy [6]. This treatment significantly improved the overall survival (OS) of CML patients and raised the possibility of disease cure [7].

Signal transduction inhibitor 571 (STI571), known better today as Imatinib (*Aka* Glivec™), a TKI, was the first targeted therapeutic drug developed for CML management [8]. Imatinib binds to the BCR-ABL1 kinase domain, which is in an inactive conformation, in a pocket designated for the ATP-binding site. This binding prevents the transfer of a phosphate group to tyrosine on the protein substrate and the consequent activation of the phosphorylated protein. As a result, leukaemic cell death is promoted due to proliferative signals being prevented from reaching the nucleus [9]. Therapeutic milestones in CML are gauged against specific molecular levels over a defined timeline, to assess optimal response and identify potential risk of treatment failure [10]. Real-time quantitative polymerase chain reaction (RQ-PCR) expressed as a BCR-ABL1 percentage (%) is the preferred method to monitor molecular response to TKI therapy.

Imatinib revolutionised CML patient survival [11]. However, a number of patients did not respond to first-line therapy with Imatinib [12]. Subsequently, five more drugs (Nilotinib, Dasatinib, Bosutinib, Ponatinib, and Asciminib) were approved for the management of CML [13, 14]. Additionally, the long-term adverse events to TKI therapy were recognised within the first decade of follow-up, hence the need to minimise TKI therapy exposure arose [15]. This knowledge led to the next step of precision management of CML patients in the form of TKI discontinuation, better known as treatment-free remission(TFR) [16–20].

Trials conducted on TFR in CML patients have met with measured success. These have led to the development of TFR management guidelines being incorporated into clinical practice [21–26]. Patients attaining a good molecular response to TKI therapy are candidates for TFR. For a patient to be considered for a TFR trial, a deep molecular response (DMR), defined as a BCR-ABL1 ≤ 0.01% (log 4 reduction in BCR-ABL1 transcripts, referred to as MR4) or ≤0.0032% (log 4.5 reduction in BCR-ABL1 transcripts, referred to MR4.5), is the primary target [21–25]. For DMR testing to be recognised and for patients to thus qualify for TFR, PCR testing for BCR-ABL1 requires an international standardised (IS) endorsed laboratory, with a turn-around time of a minimum of 1 month [24].

Other important criteria for attempting a trial of TFR include baseline chronic phase of the disease, an overall minimum period on TKI therapy of 5 years, a sustained DMR for a minimum of 2-3 years, absence of a mutation and a typical BCR-ABL1 transcript (e13a2 or e14a2) at diagnosis [21–25]. Regular testing in the initial phase of follow-up, as well as a motivated patient, are also mandatory requirements. TFR is thus fast becoming a goal of therapy in CML management [27], for those individuals who meet the predefined targets or criteria for TFR.

This is especially so, in view of the growing concern about the long-term adverse effects of TKI therapy [15]. Trials conducted thus far have described numerous variables that have a trend towards predicting successful TFR, with the most consistent being a sustained DMR and the cumulative period of TKI therapy [28, 29].

A number of smaller, single-centre studies on CML TFR [30–35] have also been reported over the years. The study numbers for patients undergoing TFR as per international and institutional guidelines were modest, ranging between 4 and 25 patients. Most of these studies report the percentage of treated CML patients at the respective institutes qualifying for TFR between 5 and 20%. The TFR success rates ranged between 56 and 72% across these studies (Table 1).

There is little data published about CML in South Africa (SA) and Africa [36–43], and no study to our knowledge, documenting the practicality and safety of TFR in a SA cohort. This study aims to contribute local data from SA on the evolving management of CML, specifically TFR.

## 2. Patients and Method

A retrospective study was designed to analyse CML patients at King Edward VIII Hospital (KEH) haematology unit attempting TFR. These patients had confirmed balanced genetic translocation, *t*(9;22)(q34;q11.2) according to the World Health Organisation (WHO) classification [44] and were in chronic phase at the time of diagnosis. The patients had also been on TKI therapy for at least 5 years and achieved a DMR for a minimum period of 3 years for first generation TKI and 2 years for second generation TKIs. Patients meeting these criteria as per European LeukemiaNet (ELN) guideline followed at KEH, as shown in Table 2. [22], attempted TKI discontinuation.

Patients attempting TFR were followed up for a minimum time period of 6 months as the greatest risk for failure of TFR is documented within this first 6-month period [45]. The information for analysis was collected from June 2020 up to May 2022. The study protocol was approved by the Ethics Committees of the University of KwaZulu-Natal (protocol number BREC/00004222/2022). The research was conducted according to the principles of the Declaration of Helsinki.

Defining the TFR success rate from TKI treatment discontinuation for KEH CML patients was the primary endpoint of the study. The secondary endpoint included factors that might predict a successful TFR trial.

Information collected on each of the patients, apart from demographic details, included exposure to interferon alpha (IFN*α*), specific TKI treatment at the time of discontinuation, reasons for discontinuation, total TKI treatment duration and molecular response, time to DMR, and period of DMR. The Sokal score, which is a prognostic score in CML, was calculated with an assessment of the patient's age, spleen size, platelet count, and peripheral blood myeloblast count at diagnosis [46]. The presenting symptoms and signs, as well as CML stage and cytogenetics, were also documented. Patients' full blood count (FBC) and differential counts, urea and electrolytes, and RQ-PCR measurement of BCR-ABL1 transcripts according to the IS ratio were also documented at baseline and during the follow-up period of treatment discontinuation. Documentation of withdrawal symptoms after TKI discontinuation, in the form of musculoskeletal pain, was recorded. Other information of interest was whether patients who failed TFR therapy were able to attain a second successful DMR after reinitiating TKI therapy. Failure of TFR was defined as a loss of major molecular response (MMR). This TFR failure was a laboratory diagnosis, with a BCR-ABL1 transcript of >0.1%, and no accompanying clinical signs.

### 2.1. Response Definitions

The patients were monitored with routine RQ-PCR for the BCR-ABL1 transcripts in an IS-accredited laboratory. Molecular responses of the RQ-PCR were expressed as a percentage. According to the ELN 2020 guidelines [22], complete cytogenetic response (CCyR) was defined as a PCR <1%, MMR as a PCR <0.1%, and a DMR as a PCR <0.01% (MR4) or <0.0032% (MR4.5). In the study cohort, the results of patients attempting TFR were monitored monthly (clinically and with molecular testing) for the first 6 months, and 2 monthly for the remainder of the first year of the TFR trial and 3 monthly beyond a year.

### 2.2. Statistical Analysis

The data were analysed using STATA software, version 17 (Statistics/Data Analysis, StataCorp LLC, College Station, Texas, United States), with numerical data reported as medians and ranges, and categorical data expressed in percentages and frequencies. Continuous variables included age at diagnosis, total duration on TKI, and duration of DMR and were analysed using a two-sample Wilcoxon rank-sum (Mann–Whitney) test. Other variables, including Sokal score, exposure to IFN*α*, and withdrawal symptoms, were analysed using Fisher's exact method.

### 2.3. Results

#### 2.3.1. Patients

**Figure 1:**
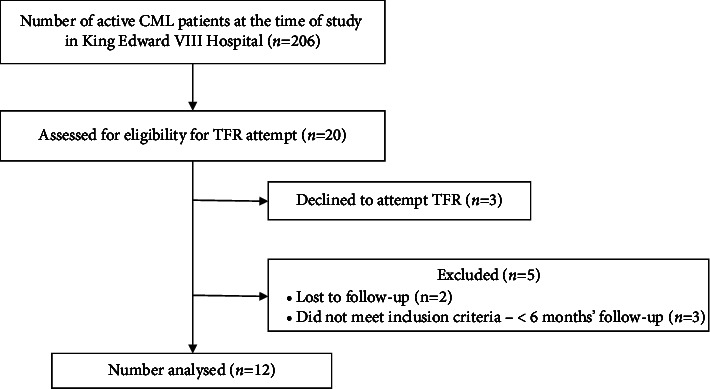
Flowchart depicting chronic myeloid leukaemia (CML) patients qualifying for treatment-free remission (TFR).

The total number of CML patients being treated in KEH at the time of the study was 206. The number of patients with CML qualifying for the TFR trial at the time of the study was 20 (10%), as shown in Figure 1.

The mean period of follow-up of the overall cohort was 13 months (SD 4.3). During the study period, 12 patients met the study inclusion criteria and had discontinued TKI therapy as per standard treatment guidelines (STG) at KEH. All 12 patients attempted TFR due to a shared decision (agreement between treating physician and patient to discontinue TKI therapy), with four of these patients having treatment-associated toxicity in addition.

The median age at disease diagnosis and at the time of discontinuation was 51 years (range 29–66) and 63 years (range 44–82), respectively, for patients attempting TFR. The patient characteristics, presenting clinical and haematological features, are reported in Table 3.

The most common symptoms that patients presented with at CML diagnosis were bleeding sequelae (33%), abdominal distension (25%), and early satiety accompanied by loss of weight (17%). Two patients presented with hyperviscosity, with one patient requiring leukapheresis. An incidental finding of CML, during routine testing, was noted in 33% of patients. All patients attempting TFR were in the chronic phase at diagnosis, with no additional cytogenetic abnormalities noted.

Of the patients attempting TFR, 11 of 12 patients (92%) were on Imatinib and one on second-line therapy with Nilotinib. This patient was on second-line treatment due to poor molecular response to front-lineImatinib therapy. Two patients were HIV positive; both remained treatment-free up to the end of the study follow-up period.

#### 2.3.2. Treatment-Free Remission and Relapse

**Figure 2:**
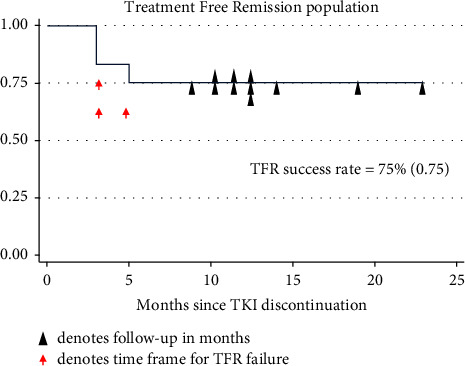
Kaplan–Meier estimates of TFR after TKI discontinuation in 12 patients with chronic myeloid leukaemia attempting TFR. The estimated survival without loss of major molecular response (failed TFR) at 12 months (media follow-up) was 75% for patients attempting TFR. TFR: treatment-free remission; TKI: tyrosine kinase inhibitor.

The overall TFR cohort showed a success rate of 75% (9 out of 12) at a median follow-up of 12 months (range [8–23]) (Figure 2). Successful TFR was defined as maintenance of MMR. There was only one patient that was on a second-generation TKI (Nilotinib), who had a successful TFR trial at 10 months of follow-up. This patient was on TKI therapy for a period of 184 months, total time in DMR of 33 months, and the time to DMR was 61 months.

Failed TFR referred to patients who lost MMR. The characteristics of the patients who failed TFR are detailed in Table 4. Of the three patients (25%) who failed TFR, all were female, and all lost MMR within the first 6 months of commencing the TFR trial. Despite the strict TFR Standard Treatment Guideline (STG) implementation, one of the patients only recommenced treatment 2 months after the loss of CCyR. She regained DMR within 6 months of TKI recommencement. The other two patients lost MMR and regained DMR within four months, upon recommencement of treatment.

**Figure 3:**
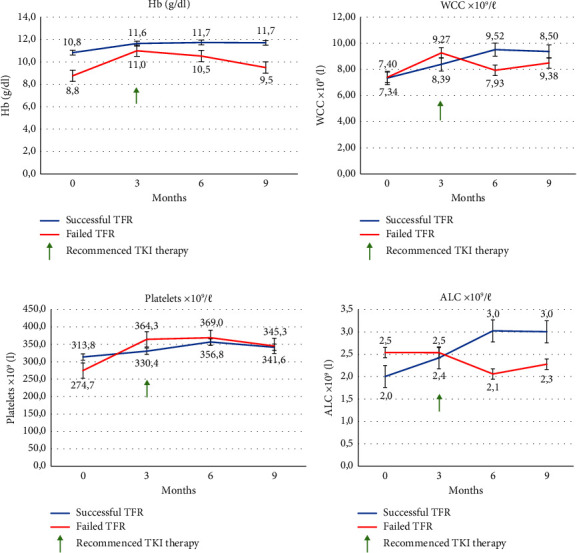
(a–d) Depicting changes in haematological parameters ((a) Hb: haemoglobin; (b) WCC: white cell count; (c) platelets; and (d) ALC: absolute lymphocyte count) in patients attempting TFR, at time points 0, 3, 6, and 9 months. Blue lines depict patients with successful TFR, and red lines depict failed TFR attempt. Green arrow depicts mean period of recommencement of TKI in the failed TFR group. TFR: treatment-free remission; TKI: tyrosine kinase inhibitor.

Upon embarking on TFR trial, there were improvements in all haematological laboratory parameters compared to baseline values over time (Figure 3) in all patients. Improvements in haemoglobin (*p* = 0.02) at 3 months, WCC (*p* = 0.001) at 6 months, platelets (*p* = 0.03) at 6 months, and absolute lymphocyte count (ALC) (*p* = 0.60) at 6 months were noted in patients with successful TFR. There was a notable drop in all four parameters with the recommencement of TKI in the failed TFR cohort. Changes in the ALC among patients who had a successful TFR showed a trend towards significance (*p* = 0.06), as opposed to patients who failed TFR (*p* = 0.60) and who had no significant change in ALC. There was no incidence of disease transformation in the study population.

### 2.4. Factors Influencing TFR

The results for the various factors predicting TFR successes are shown in Table 5. The median total period of TKI therapy in the successful group and unsuccessful group was 137 months (range 87–192) and 96 months (range 87–97), respectively, which differed significantly (Mann–Whitney *U* = 27, *p* <  0.05 two-tailed). The rest of the parameters showed no significance in terms of predicting outcome.

## 3. Discussion

The approach to CML management, with planned lifelong TKI therapy due to improved OS [7], has changed over the last 4 years. Numerous TFR trials have been undertaken in clinical practice globally [30–35] since the publication of treatment guidelines [21–25]. However, there is little data on CML from South Africa, and even less on TFR [38–41].

Although small, this single-centre study reaffirmed that TFR is safe, with no disease progression and a TFR success rate of 75% at 12 months of follow-up. Whilst the success rate is superior to those described in landmark studies [16–20] as well as other single-centre studies (Table 1), the follow-up period was notably shorter in our study, at most half the time. Another recently published, single-centre study reported in Kuwait by Alhuraiji et al. [47] also noted a high TFR success rate (74%), which they attributed to a prolonged period of DMR (>5 years). Despite the rate of TFR success being 75% at 12 months, and the risk of failing TFR being significantly less after 6 months [24], late relapses have been reported [48]. So the relatively shorter period of study follow-up, as well as small numbers in our study, may be confounders to the true long-term rate of TFR success in our CML population and may likely change with time.

The percentage of patients, who qualified for TFR, in our study (10%), was significantly less compared to the theoretical percentage (40%), as described by Goldberg and Hamarman of patients who would be eligible for TFR on first-line treatment [27]. The latter theoretical estimate, however, is not a true reflection of real-world data. The percentage reported (5–20%) across the globe in the single-centre studies [30–35] is a more realistic expectation for CMLpatients qualifying for TFR.

Of the biological factors as potential positive predictors of TFR success [29] from those reported in other studies, the only factor that had statistical significance, in our study, was the total time on TKI therapy [17, 18]. The median total time on TKI for our cohort was almost 1.5 to 4-fold more than the recommended minimum total period on TKI as per guidelines before discontinuation of TKI [21–25], which may also be the reason for the higher rate of success in our cohort. The cumulative period on TKI has been shown to be one of two strong predictors of TFR success [17, 18, 47]. In a study recently presented at ASCO by the MD Anderson group, Haddad et al. [49] noted higher rates of success (79%) for patients who remained in DMR for more than 5 years, which was not noted to be significant in our analysis. All other factors conferring influence on outcomes described in previous studies were not considered significant viz. Sokal score [16], withdrawal symptoms [17], older age [19], and prior exposure to IFN alpha [16]. Our study mirrored the latter study findings, with none of the aforementioned variables exhibiting any statistically significant influence on TFR success in our KEH cohort as well.

The changes noted in haematological parameters were assessed and appeared to show a general increase among both the group of successful and unsuccessful patients undergoing TFR. An interesting difference was, however noted, related to the ALC. This ALC appeared to show a trend towards significance in successful TFR patients, as opposed to patients who failed TFR (*p* = 0.60). The latter may potentially be an indicator of the increased NK cell subsets described, by Imagawa et al. in the Dasatinib Discontinuation study (DADI) trial [20] and the Spanish study by Vigón et al. [50]. Further studies to verify NK cell subsets influence over successful TFR were reported by Hseih [51] and Irani et al. [52] in two separate studies. The ALC, which in addition to enumerating B- and T-lymphocytes, also includes NK cells, and the higher NK cells may predict successful TFR in CML. This should ideally be evaluated prospectively to determine whether ALC can be adopted as a surrogate biomarker to predict TFR success for patients undergoing TFR and whether it may be a quicker and more cost-effective method of predicting successful outcomes.

The one patient who failed TFR attempt, and subsequently regained DMR, resumed treatment beyond the advised period of reinitiation (with loss of CCyR) raises the question as to whether the stringency in monitoring in CML TFR can potentially be relaxed. Jumaa et al. [35] performed PCR's on their 18-patient cohort only 3 monthly within the first 6 months but reported no adverse events in the form of disease resistance or transformation for those patients who failed TFR. The latter study had a low patient number and probably will require a larger prospective randomised controlled trial before suggestions can be made for adjustment to the current TFR guidelines.

Patients undertaking TFR under non stringent conditions were not included in the analysis of our study. A few CML patients on TKIs from KEH underwent TFR trial under non stringent conditions. One patient attempted TFR because of pregnancy and the risk posed to the foetus by TKI therapy (Dasatinib) in pregnancy, despite having a mutation (Y253H—resistant to Imatinib and Nilotinib). A mutation is considered a contraindication to attempting TFR in CML [21–25], as the risk for clonal selection and disease progression is high [53]. This patient had achieved a DMR at the time of discontinuing TKI, but failed TFR trial, and lost CCyR off TKI after 6 months of discontinuing TKI. Postpartum, she regained DMR after 3 months of reinitiating TKI (Dasatinib) and remained in DMR after 13 months of follow-up. A small retrospective analysis, by Claudiani et al. involving patients with kinase domain mutations (KDM) [54], did not report any adverse events during TFR, and a 50% TFR success rate. The latter analysis did concede that patients with a KDM may attempt TFR due to significant treatment-related toxicity. However, this needs a larger prospective trial to assess TFR probability and safety in this cohort. The second patient did not follow-up (5 PCR tests only) strictly according to the institutional guidelines, due to defaulting, but remained in DMR after 20 months' follow-up. The last patient attempted TFR after being in DMR for only 6 months and 24 months of the total period on TKI therapy (since diagnosis), attempted TFR of her own accord, and failed TFR attempt at 6 months. She regained DMR after 6 months of recommencement of TKI therapy.

There can be no compromise to patient care or patients undertaking TFR beyond currently stipulated guidelines, as highlighted by the latter two patients. Non stringent TFR attempts may be offered to patients under exceptional circumstances, e.g., pregnancy or due to significant toxicities secondary to long-term TKI use. Should patients attempt TFR for indications beyond the current guidelines, they should carefully weigh-up the risk vs the benefit, as well as be counselled about compliance with strict PCR monitoring. The responses for these non-prescribed TFR case reports, may however, add to the current indication for CML TFR.

## 4. Conclusion

The goalpost of CML has been repositioned, with TFR being incorporated into mainstream clinical practice. No definitive guidelines can be drawn from our study due to the small patient numbers. A larger prospective multicentre study needs to be performed in Africa, as this may impact dramatically on disease burden and economic benefit to both patients and the healthcare systems. The latter requires adequate molecular monitoring and prompt reinitiation of treatment. This study has shown this goal of TFR to be potentially attainable in Africa and paves the way for further prospective study.

## Figures and Tables

**Table 1:** Small single-centre studies on treatment-free remission in CML.

Study	Kong et al. WCI [30]	Cerveira et al. POI [31]	McMullan et al. BCC [32]	Benjamini et al. MDAH [33]	Lino et al. YPCH [34]	Jumaa et al. BCOH [35]
No. of pt with CML	206	Not reported	105	Not reported	85	Not reported
No. of pt attempting TFR	25	25	21	35 (8)	21 (4)	18
% of pt qualifying for TFR	12.1%	N/A	20%	16%	5%	N/A
No. of months on TKI	N/A	100		96 [8–136]	68 [22–161]	93
No. of months in DMR	N/A	41		63 [1–106]	44 [16–96]	37
Median follow-up after TFR attempt in months	42 [15–154]	24		16 [2–106]	32 [18–69]	24
% success of TFR	56%	58%		59%	67%	72%

*Patients who failed TFR attempt*						
Median period to restarting TKI in months [range]	2.7 [2.7–24]	5 [4–9]		3.5 [1–32]	5 [1–6]	9 [3–12]
Median period to regaining DMR in months [range]		5 [2–10]		6 [2–18]	3	3

CML: chronic myeloid leukaemia; TFR: treatment-free remission; TKI: tyrosine kinase inhibitor; DMR: deep molecular response; WCI: Winship Cancer Institute; POI: Portuguese Oncology Institute; BCOH: Basra Centre for Oncology and Hematology; BCC: Belfast City Hospital; MDAH: MD Anderson Houston; YPCH: Yamanashi Prefectural Central Hospital; No.: number; pt: patient.

**Table 2:** European LeukemiaNet requirements for tyrosine kinase inhibitor discontinuation [22].

Mandatory test results	(i) CML in first CP only (data are lacking outside this setting)
	(ii) Motivated patient with structured communication
	(iii) Access to high-quality quantitative PCR using the international scale (IS)
	(iv) Rapid turn-around of PCR
	(v) Patient's agreement to more frequent monitoring after stopping treatment: monthly for the first 6 months, every 2 months for months 7–12, and every 3 months thereafter

Minimal (stop allowed)	(i) First-line therapy or second-line, if intolerance was the only reason for changing TKI
	(ii) Typical e13a2 or e14a2 BCR-ABL1 transcripts
	(iii) Duration of TKI therapy >5 years (>4 years for 2G-TKI)
	(iv) Duration of DMR (MR4 or better) > 2 years
	(v) No prior treatment failure

Optimal (stop recommended for consideration)	(i) Duration of TKI therapy >5 years
	(ii) Duration of DMR >3 years if MR4

CML: chronic myeloid leukaemia; CP: chronic phase; PCR: polymerase chain reaction; TKI: tyrosine kinase inhibitor; DMR: deep molecular response; MR4: molecular response with log 4 reduction in BCR/ABL transcripts.

**Table 3:** Profile of patients attempting treatment-free remission.

	Overall group	Successful	Unsuccessful
Number	12	9	3
Median age at diagnosis in years (range)	51 (29–66)	50 (29–66)	53 (45–59)
Median age at discontinuation in years (range)	63 (44–82)	63 (44–82)	61 (52–67)
Gender, *n* (%)			
Males	3 (25)	3 (33)	0
Female	9 (75)	6 (67)	3 (100)
Sokal score, *n* (%)			
Low	6 (50)	5 (56)	1 (33)
Intermediate	1 (8)	1 (11)	N/A
High	3 (25)	1 (11)	2 (67)
Indeterminate	2 (17)	2 (22)	N/A
Median time of follow-up in months (range)	12 (8–23)	12 (8–23)	12 (11–14)
TKI at discontinuation, *n* (%)			
Imatinib	11 (92)	8 (89)	3 (100)
Nilotinib	1 (8)	1 (11)	0
Reasons for TKI discontinuation, *n* (%)			
Shared^∗^	12 (100)	9 (75)	3 (25)
Toxicity	4 (33)	2 (17)	2 (17)
Median total duration of TKI in months (range)	133 (87–192)	137 (87–192)	96 (87–97)
Median time to DMR in months (range) overall	39 (13–89)	42 (13–89)	28 (13–54)
MR4 (*n* − 2)	72 (54–89)	89	54
MR4.5 (*n* − 10)	32 (13–61)	36 (13–61)	15 (13−17)
Median duration of DMR in months (range) overall	87 (33–148)	99 (33–148)	70 (43–82)
MR4 (*n* − 2)	40 (37–43)	37	43
MR4.5 (*n* − 10)	95 (33–148)	99 (33–148)	76 (70–82)

TKI: tyrosine kinase inhibitor; DMR: deep molecular response; TFR: treatment-free remission; shared decision^*∗*^: agreement between treating physician and patient to discontinue TKI therapy.

**Table 4:** Characteristics of patients failing treatment-free remission.

Parameter		Overall	Patient 1	Patient 2	Patient 3
TFR failure, *n*^*∗*^ (%)		3^*∗*^ (25)	—	—	—
Follow-up after TKI discontinuation in months, median [range]		13 [9–14]	9	13	14
Time to molecular relapse in months, median [range]		3 [3–4]	3	4	3
Time to recommencement of TKI therapy in months, median [range]		2 [1–2]	1	2	2
Time to MMR after molecular relapse in months, median [range]		1 [1–3]	1	1	3
Time to DMR after failed TFR in months, median [range]		4 [2–6]	2	4	6

Response at last follow-up, *n* (%)	MR4.0	1 (33)			*X*
	MR4.5	2 (67)	*X*	*X*	

TKI: tyrosine kinase inhibitor; DMR: deep molecular response; MR4.5: ≥4.5-log reduction in BCR-ABL1 transcripts from baseline; MR4.0: ≥4.0-log reduction in BCR-ABL1 transcripts from baseline; MMR: major molecular response. ^*∗*^Total TFR cohort = 12 patients.

**Table 5:** Factors predicting treatment-free response outcome.

Number	Successful TFR *n* = 9	Failed TFR *n* = 3	*p* value
Age at diagnosis in years, median [range]^†^	50 [29–66]	53 [45–59]	0.60
Total duration on TKI in months^†^, median [range]	137 [87–192]	96 [87–97]	0.02
Duration of DMR in months^†^, median [range]	99 [83–113]	70 [43–82]	0.16
Sokal score, *n* (%)^*∗*^			0.50
Low	5 (56)	1 (33)	
Intermediate	1 (11)	N/A	
High	1 (11)	2 (67)	
Indeterminate	2 (22)	N/A	
Exposure to IFN*α, n* (%)^*∗*^			0.90
No	7 (78)	3 (100)	
Yes	2 (22)	0 (0)	
Withdrawal symptoms, *n* (%)^*∗*^			0.50
No	3 (33)	2 (67)	
Yes	6 (67)	1 (33)	

^†^Two-sample Wilcoxon rank-sum (Mann–Whitney) test; ^*∗*^Fisher's exact; IQR: interquartile range; MR: deep molecular remission; IFN*α*: interferon alpha; TKI: tyrosine kinase inhibitor.

## Data Availability

The data supporting the current study are available from the corresponding author upon request.
